# The habenula governs the attribution of incentive salience to reward predictive cues

**DOI:** 10.3389/fnhum.2013.00781

**Published:** 2013-12-09

**Authors:** Carey L. Danna, Paul D. Shepard, Greg I. Elmer

**Affiliations:** Department of Psychiatry, Maryland Psychiatric Research Center, School of Medicine, University of MarylandBaltimore, MD, USA

**Keywords:** sign-tracking, goal-tracking, mesolimbic, dopamine, autoshape, motivation

## Abstract

The attribution of incentive salience to reward associated cues is critical for motivation and the pursuit of rewards. Disruptions in the integrity of the neural systems controlling these processes can lead to avolition and anhedonia, symptoms that cross the diagnostic boundaries of many neuropsychiatric illnesses. Here, we consider whether the habenula (Hb), a region recently demonstrated to encode negatively valenced events, also modulates the attribution of incentive salience to a neutral cue predicting a food reward. The Pavlovian autoshaping paradigm was used in the rat as an investigative tool to dissociate Pavlovian learning processes imparting strictly predictive value from learning that attributes incentive motivational value. Electrolytic lesions of the fasciculus retroflexus (*fr*), the sole pathway through which descending Hb efferents are conveyed, significantly increased incentive salience as measured by conditioned approaches to a cue light predictive of reward. Conversely, generation of a fictive Hb signal via *fr* stimulation during CS+ presentation significantly decreased the incentive salience of the predictive cue. Neither manipulation altered the reward predictive value of the cue as measured by conditioned approach to the food. Our results provide new evidence supporting a significant role for the Hb in governing the attribution of incentive motivational salience to reward predictive cues and further imply that pathological changes in Hb activity could contribute to the aberrant pursuit of debilitating goals or avolition and depression-like symptoms.

## INTRODUCTION

Cues and imagery associated with rewards can evoke pleasure, motivate approach, and lead to reward consumption. The integrity of the motivational systems controlling these processes are critical to an individual’s functional capacity and quality of life; disruptions can lead to amotivation, avolition, and anhedonia, symptoms that cross the diagnostic boundaries of many neuropsychiatric disorders (Insel et al., 2010; Cohen et al., 2012; Morris and Cuthbert, 2012). While an imbalance in the opposing constructs mediating approach and avoidance could contribute to the development of these clinical symptoms, avoidance fails to faithfully capture the clinical features of anhedonia and avolition. Rather, these maladaptive behaviors could reflect functional changes in systems encoding the incentive salience of stimuli predicting reward. For example, increased attribution of incentive salience to reward cues could lead to reduced impulse control and the aberrant pursuit of rewards while a failure to appropriately assign incentive salience to reward cues is likely to diminish motivation and volition.

The Pavlovian autoshaping paradigm is a method well suited to investigate the circuitry underlying attribution of incentive salience (Brown and Jenkins, 1968; Gamzu and Williams, 1973; Pellegrini et al., 2008). In this procedure, repeated presentation of a stimulus prior to reward delivery results in a quantifiable and progressive increase in conditioned approach toward the goal (e.g., food) as the predictive value of the cue is learned. This conditioned response is termed “goal-tracking.” A quantifiable and progressive increase in conditioned approach to the stimuli can also occur as it is increasingly endowed with incentive salience. This conditioned response is termed “sign-tracking.” The paradigm offers the possibility of separately assessing predictive value from the motivational salience associated with cues predicting reward delivery. Recent studies demonstrate that dopamine (DA) transmission is required for sign-tracking but not for goal-tracking (Day et al., 2007; Danna and Elmer, 2010; Flagel et al., 2011b).

The circuitry responsible for producing changes in DA cell activity associated with the attribution of incentive salience is incompletely understood. Considerable interest has developed in the role of the lateral habenula (LHb) and rostral medial tegmentum (RMTg) in mediating the transient suppression in DA cell firing associated with negative reward prediction errors (Hong et al., 2011). LHb neurons are coherently activated by the absence of expected rewards (Matsumoto and Hikosaka, 2009) and are presumed to mediate their influence on DA cell firing via connections with GABAergic RMTg neurons (Hong et al., 2011). In addition to its functional role in encoding negatively valenced associations, increased LHb activity during reward anticipation (Bromberg-Martin et al., 2010), and in animals predisposed to assigning motivational properties to neutral cues (Flagel et al., 2011a) suggest a role in governing the attribution of incentive salience. To date, this possibility has not been systematically explored.

In the present series of experiments, electrolytic lesions and electrical stimulation of the fasciculus retroflexus (*fr*), the sole pathway through which descending LHb efferents are conveyed, were used to assess the contribution of this pathway to specific components of the Pavlovian autoshaping procedure. Our results provide new evidence supporting a significant role for the Hb in governing the attribution of incentive motivational salience to reward predictive cues.

## MATERIALS AND METHODS

### SUBJECTS

Adult, male, Sprague-Dawley rats (Charles River Laboratories, Wilmington, MA, USA) weighing approximately 225–250 g at the start of the experiment were used as experimental subjects. Rats were housed in a temperature controlled vivarium under a 12:12 h light:dark cycle and provided unrestricted access to food prior to the start of the behavioral experiments. All studies were conducted in strict accordance with the principals outlined in the NIH Guide for Care and Use of Laboratory Animals and were sanctioned by the Institutional Animal Care and Use Committee of the University of Maryland, Baltimore.

### PAVLOVIAN AUTOSHAPING PROCEDURE

Six rat operant chambers (dimensions 25 cm L × 21 cm W × 20 cm H; Med Associates, St. Albans, VT, USA) under the control of Med Associates software were used during the completion of these experiments. Each chamber was equipped with two custom cue lights consisting of a translucent 6 × 6 cm panel positioned on either side of a pellet delivery system. One light in each chamber was covered with translucent blue plastic to enhance the difference between visual cues. A pellet delivery system provided a single 45 mg sucrose pellet to a small recessed receptacle located between the two cue lights. Entrances into the pellet receptacle and approaches to each cue light were detected by separate infrared photobeam detectors. Rats were required to be within 2 cm of the cue light to interrupt the beam. A retractable lever was positioned in the middle panel at the opposite end of the chamber from the stimulus lights and pellet receptacle.

Subjects were maintained at 85% of their free feeding weight by rationing daily food allotment. For nine consecutive days, rats were placed in an operant chamber for a single session consisting of 30 trials in which a single CS+ or CS- cue was presented pseudo-randomly for a variable interval of 20 sec (±10 s).

Three experiments were conducted using the same autoshape procedure, two using *fr* lesioned animals and one using electrical stimulation (see **Table 1**). *fr Lesion*. In one group, a full contrast conditioning schedule was used; subjects were reinforced 100% of the time the CS+ was presented and 0% when the CS- was presented. A separate group of *fr* lesioned rats, a partial contrast conditioning schedule was used (henceforth termed CS^33^). Subjects were reinforced 100% of the time when the CS+ was presented and 33% of the time when the alternate CS was presented. *Electrical stimulation*. Only the full contrast schedule was used in the electrical stimulation study. In all conditions, a retractable lever was presented at the rear of the chamber thirty sec after stimulus presentation. The rat was required to press the lever in order to advance to the next trial ensuring that the animal was equidistant from both cues at the time of presentation (Parkinson et al., 2000).

**Table 1:** Experimental conditions for lesion and electrical stimulation studies.

Experiment	Groups	Sub-groups	*n*
*fr* Lesion full contrast	Sham		10
	Lesion		8
*fr* Lesion partial contrast	Sham		6
	Lesion		7
Stimulation	Stim during CS+	Stim	7
	Stim during US	Stim	8
		Sham	8

### ELECTROLYTIC LESIONS OF THE FASCICULUS RETROFLEXUS

Prior to the start of autoshape training, two groups of rats were anesthetized with ketamine (80 mg/kg, i.p.) and xylazine (10 mg/kg, i.p.) and mounted in a stereotaxic apparatus using atramatic ear bars. The scalp was incised and two burr holes drilled on both sides of the skull overlying the caudal diencephalon (AP: -4.56 mm from Bregma, ML: 0.5 mm from midline, Paxinos and Watson, 2007). A concentric bipolar stimulating electrode (SNEX-100, Rhodes Medical Instruments) was lowered into the region of the *fr* (7.3 mm below pial surface) and a DC current (0.5 mA) was applied for 15 s using a constant current stimulator. These parameters were empirically derived to produce a lesion restricted to the diameter of the *fr*. Electrolytic *fr* lesions were made consecutively on each side of the brain. Sham control animals underwent an identical surgical procedure with the exception that no current was passed through the stimulating electrode. Four days following surgery, rats entered the behavioral arm of the study.

### STIMULATION OF THE FASCICULUS RETROFLEXUS

A separate group of rats was prepared for surgery and implanted with stimulating electrodes in the *fr* in a manner identical to that described above for the sham control group. Once positioned, the electrodes were affixed to the skull using DenMat dental cement (Santa Maria, CA, USA). Four screws, affixed to parietal and frontal skull, served as anchors. Rats recovered in their home cages for 4 days prior to entering the behavioral arm of the study. Prior to the start of each session, rats were connected to a Med Associates PHM-152 stimulator. Electrical stimulations consisted of four 3-s trains of rectangular constant current pulses (0.25 mA; 3 Hz) and were applied during the CS+ presentation (CS+ Stim) or simultaneously with reward delivery (US Stim). A concentric bipolar stimulating electrode (SNEX-100, Rhodes Medical Instruments) was used to deliver stimulation. Stimulation parameters were based upon the approximate spontaneous firing rate of the Hb projection neurons (between 2.2 and 4.3 Hz; Weiss and Veh, 2011) and results demonstrating significant DA inhibition in the VTA and substantia nigra (97%; Ji and Shepard, 2007). A smaller current was used in the present study (0.25 vs. 0.5 mA) to confine the spread of electrical stimulation. Repeated stimulation with a longer train was used to suppress DA neurons for a longer duration of the CS or US presentation. The Sham control rats were attached to the swivel but were not connected to the stimulator. CS+ Stim, US Stim, and Sham groups were run concurrently to allow joint comparison with the Sham controls.

#### Histology

Following the completion of experimental procedures, animals were euthanized and the brains were removed, sectioned, and stained with cresyl violet for verification of lesion and electrode placements. In order to be included in the statistical analysis, lesions were required to ablate at least 25% of the *fr* on a given side and, at least 75% of the combined bilateral area of the *fr*.

#### Analysis

During the session, approach to the reward (US) and cue lights (CS) were quantified by photobeam breaks. Approach to the reward dispenser during the CS+ presentation is a conditioned response that reflects anticipation of reward delivery, referred to herein as goal-tracking. Approach to the CS+ cue during its presentation is a conditioned response that reflects the transfer of incentive motivational value to the predictive cue referred to herein as sign-tracking. The number of approaches to the CS+ and CS- lights during illumination and the difference score (CS+ approaches minus CS- approaches) as well as the number of beam breaks within the reward dispenser during CS+ and CS- presentation, and the corresponding difference score, were used as dependent variables. The number of approaches and difference scores were analyzed using one-, two-, or three-way repeated measures analysis of variance (RMANOVA) as appropriate. Tests for unequal variance and all statistical analyzes were performed using JMP software (Cary, NC, USA).

## RESULTS

### *fr* LESION

#### Full contrast conditioning schedule

**FIGURE 1:**
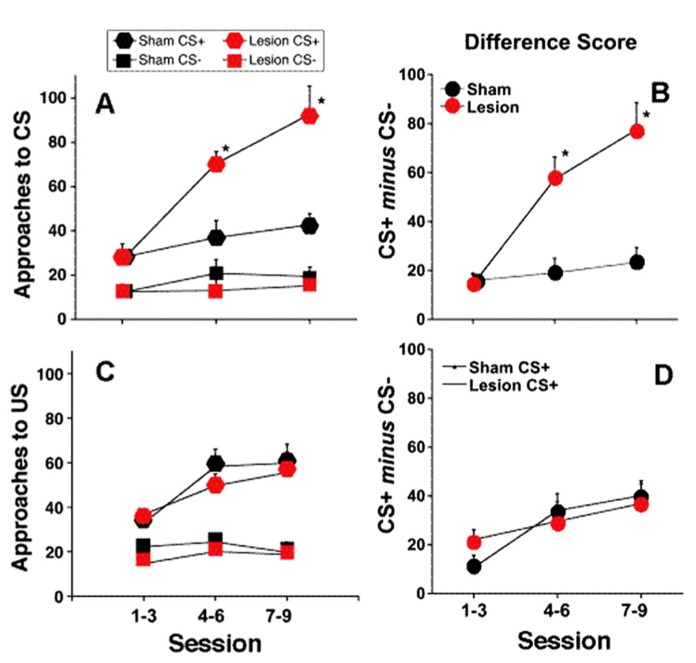
**Full contrast conditioning schedule.** **(A–D)** represent the effects of *fr* lesions on conditioned approach to each CS **(A)** and to the US **(C)** during the CS+ and CS- presentation. **(B,D)** represent the difference scores (CS+ minus CS-) derived from values represented in **(A,C)**. *n* = 10 and 8 for the Sham and Lesion groups, respectively. Error bars represent S.E.M. *p < 0.01.

Sham and lesioned animals learned the association between stimulus presentation and reward delivery (**Figure 1**). In both experimental groups, repeatedly presenting the CS+ cue prior to reward delivery resulted in a gradual increase in goal-tracking and sign-tracking during presentation of the CS+ compared to the CS- (*F*(session × cue) = 9.9; df = 2,21, *p* = 0.009). Individual subjects were equally likely to exhibit goal-tracking as sign-tracking.

*Sign-tracking:* The effects of *fr* lesion on conditioned approach to the CS+ and CS- as well as the difference score (CS+ approach minus CS- approach) is presented in **Figure 1**, panels A and B, respectively. *fr* lesion significantly increased sign-tracking compared to the sham group (CS+: *F*(lesion) = 18.44, df = 1,11, *p* = 0.002). Importantly, the number of approaches to the CS+ cue was similar during the first several sessions; differences between the two groups increased as they gained more exposure to CS+: US pairings (CS+: *F*(lesion × session) = 10.75, df = 2,10, *p* = 0.003). The sham and lesion groups did not differ in their approach to the CS-.

*Goal-tracking:* Both groups of animals learned that the CS+ predicted reward delivery as demonstrated by increased goal-tracking during the CS+ presentation across training sessions (*F*(session) = 11.57; df = 2,17, *p* = 0.0007; **Figures 1C,D**). Neither the absolute number of entries into the pellet dispenser during the CS+ or CS- presentation nor difference score was altered by *fr* lesion.

**FIGURE 2:**
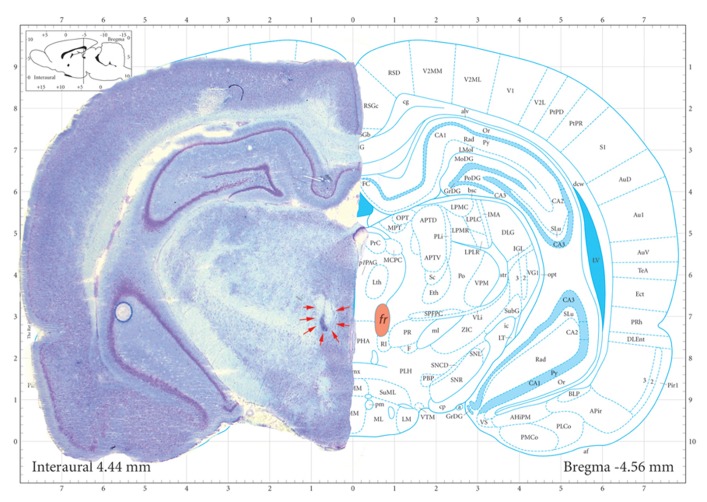
**Representative photomicrograph of an *fr* lesion.** Photomicrograph of section stained with cresyl violet. Electrolytic *fr* lesions were made consecutively on each side of the brain. Orange shaded region on the right highlights *fr* location and the arrows on outline the perimeter of the electrolytic region. Sham control animals underwent an identical surgical procedure with the exception that no current was passed through the stimulating electrode. Coronal section from Paxinos and Watson (2007).

**Figure 2** shows a representative photomicrograph of an *fr* lesion. Additional analysis demonstrates a functional relationship between lesion size and behavioral consequence. When animals that did not meet lesion criteria were considered (partial lesion but less than criteria), there was a significant correlation between number of approaches to the CS+ during the last three sessions of the training period and the extent of the *fr* lesion (*n* = 14 total; *r*^2^ = 0.32; *p* = 0.03). There was no correlation between the extent of the *fr* lesion and the number of approaches to the CS- or conditioned approach to the pellet receptacle. Overall, histological evidence of lesion, significant behavioral consequence of sham vs. lesion, and significant correlation between lesion size and behavioral are all supportive evidence for the argument that amperages were sufficient to affect the *fr*. Interestingly, a 25% lesion to the outer portion of the *fr* would suggest destruction of primarily LHb output since the lateral habenular efferents compose the mantle portion of the bundle (Herkenham and Nauta, 1979). This would explain why a partial *fr* lesion would be effective since it would constitute a much larger portion of the reward-relevant LHb. Furthermore, since there is contralateral innervation of midbrain structures from the LHb, a partial lesion would have bilateral influence.

#### Partial contrast conditioning schedule

**FIGURE 3:**
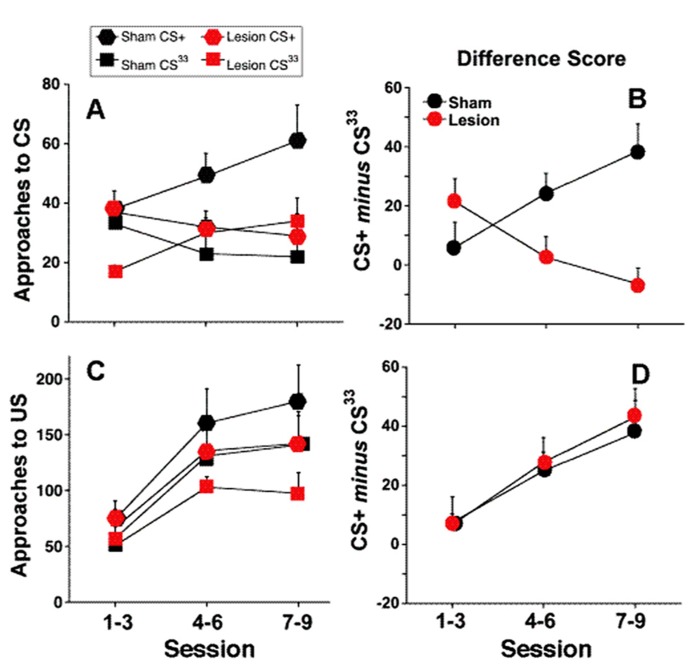
**Partial contrast conditioning schedule: (A,B) represent the effects of *fr* lesion on conditioned approach to each CS **(A)** and to the US **(B)** during the presentation of a 100% CS+ and 33% CS- presentation.** **(C,D)** represents the difference score (CS+ minus CS^33^) derived from values represented in **(A,B)**. *n* = 6, 7 for the Sham and Lesion groups, respectively. Error bars represent S.E.M.

Introduction of a partially reinforced CS (33%; CS^33^) had a marked effect on sign-tracking (**Figure 2**). Unlike the full contrast schedule, sham treated rats were initially unable to distinguish between the CS+ and CS^33^, approaching both with equal frequency during the first three sessions of training. However, over the course of the next six sessions, sham-treated rats showed a greater number of approaches to the CS+ than the CS^33^ (*F*(CS+ vs. CS^33^) = 5.83; df = 1, 12, *p* = 0.03), reflecting the assignment of increased salience to the cue paired with a more certain outcome (**Figures 3A,B**). In contrast to the sham-treated rats, *fr* lesioned animals were clearly able to discriminate between CS+ and CS^33^ during the first three sessions of training (*F*(CS+ vs. CS^33^) = 7.52; df = 1, 5, *p* = 0.04), showing a twofold greater increase in the number of approaches to the CS+ than the CS^33^. However, during subsequent sessions, rats in the *fr* lesioned group approached the CS^33^ with increasing frequency while approaches to the CS+ showed a trend toward a decline as demonstrated by a significant reduction in the difference score to near zero (*F* = 7.81; df = 2,4, *p* = 0.04; **Figure 3B**). Divergent response of sham and lesioned groups to partial contrast conditioning was further evidenced statistically by a clear divergence in their difference scores as reflected in a significant interaction as a function of training session (difference score: *F*(lesion × session) = 6.71; df = 2,10, *p* = 0.01; **Figure 3B**). Notably, no group differences were observed in the conditioned approach to the pellet dispenser (**Figures 3C,D**).

### *fr* STIMULATION

**FIGURE 4:**
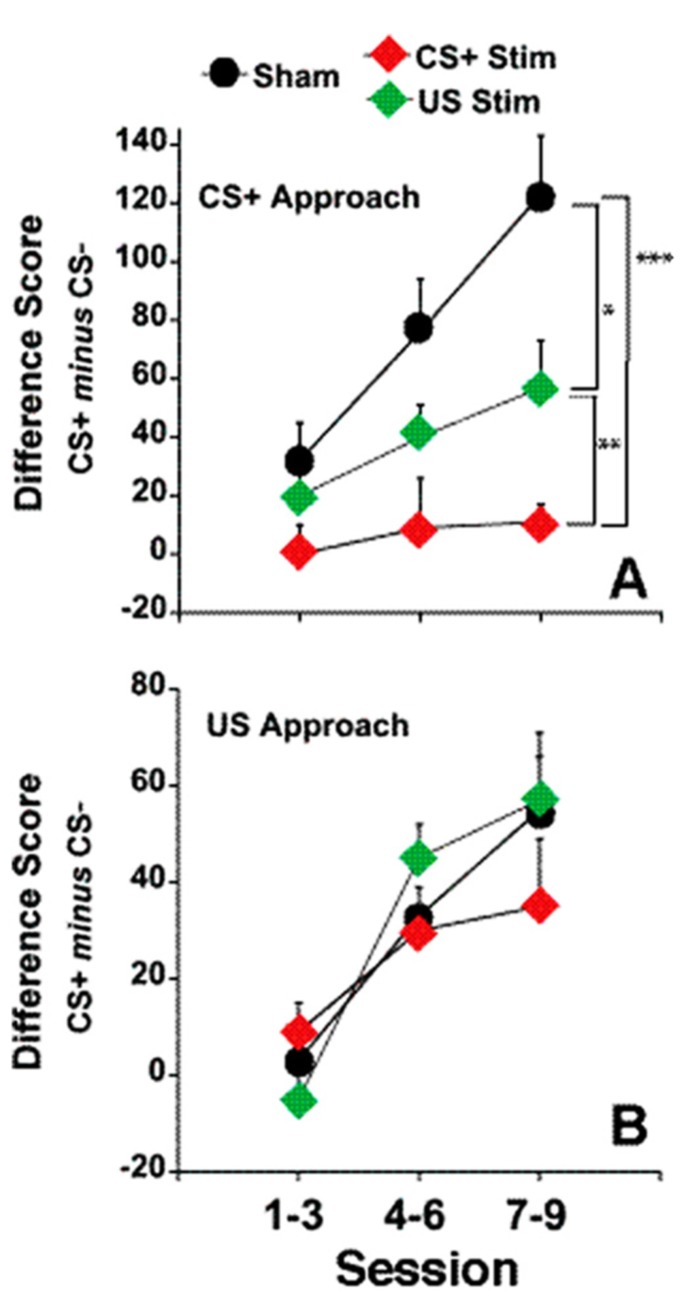
**Electrical stimulation of the *fr* during CS+ or US presentation.** **(A)** represents the effects of *fr* stimulation during CS+ presentation on conditioned approach to the CS+ vs. the CS- as described by the Difference Score (same dependent variable as seen in **Figures 1** and **2**). **(B)** represents the effects of *fr* stimulation during US presentation. *n* = 8, 7, 8 for the Sham, CS+ Stim and US Stim groups. Error bars represent S.E.M. Difference between experimental groups: ***p < 0.001, **p < 0.01, *p < 0.05.

There was no effect of *fr* stimulation on approach to the CS-, therefore, in order to simplify the presentation, only the difference score (number of CS+ approaches minus the number of CS- approaches) is shown in **Figure 4**, panels A and B.

*Sign-tracking*: A two way repeated measures ANOVA contrasting the difference score in the three groups (CS-Stim, US-Stim and Sham across training sessions) revealed a significant main effect of stimulation group (*F*(stimulation) = 10.01, df = 2,20, *p* = 0.001). Post hoc analysis of pair-wise comparisons (CS+ vs. Sham, CS+ vs. US and Sham vs. US using two way repeated measures ANOVA) shows that stimulation during CS+ decreased sign-tracking significantly from the sham group overall (*F*(stimulation) = 17.36 df = 1,13, *p* = 0.001) and as a function of session (*F*(stimulation × session) = 5.30, df = 2,12, *p* = 0.022). Stimulation during US, while decreasing sign-tracking less dramatically, also reached statistical signficance (*F*(stimulation) = 4.87, df = 1,14, *p* = 0.045) but did not differ as a function of training session. This comparison was the only contrast in which statistical analysis of the difference score and the number of conditioned approaches to the CS+ did not reach the same conclusion; conditioned approach to the CS+ was marginally significant between the two groups (*F*(stimulation) = 3.32, df = 1,13, *p* = 0.08). The contrast between the CS+ stimulation group and US stimulation group reveals a significant difference between the two conditions (*F*(stimulation) = 7.35, df = 1,13, *p* = 0.01). Except where noted above, the statistical analysis of the difference score and conditioned approach to CS+ yeild the same conclusions. Overall, these findings suggest that disruption of incentive valuation was slightly more sensitive to stimulation during the CS+ than during US.

*Goal-tracking:* Conditioned approach to the reward dispenser was not altered by *fr* stimulation (**Figure 4B**). Both groups learned the predictive association between CS+ presentation and reward presentation.

## DISCUSSION

A solid body of evidence establishes a key role for the Hb in processing aversive events and error signaling (Hikosaka, 2010; Jesuthasan, 2012; Shelton et al., 2012; Brown and Shepard, 2013; Jhou et al., 2013). The capacity of the Hb for bidirectional signaling (Matsumoto and Hikosaka, 2007) and cFos activation during incentive motivational learning (Flagel et al., 2011a) suggested a possible role in encoding positively valenced events such as reward learning and the attribution of incentive salience. This proposition was empirically tested in two complimentary studies. Eliminating the sole output of the Hb via *fr* lesion significantly increased the attribution of incentive salience to a reward predictive cue as evidenced by increased sign-tracking. Conversely, producing a fictive Hb signal via *fr* stimulation significantly decreased the attribution of incentive salience to the reward predictive cue. Neither manipulation altered reward predictive learning as measured by goal-tracking. These results provide new evidence supporting a role for the Hb to serve as a governor, in the sense of automatic control or limitation, under conditions leading to the attribution of incentive salience.

The Hb can negatively modulate phasic DA release in brain areas centrally involved in reward processing (e.g., nucleus accumbens, NAc, medial prefrontal cortex Lecourtier et al., 2008). Therefore, loss of Hb output might be expected to enhance the phasic DA response to CS+ presentation and in turn increase the attribution of incentive salience to reward predictive cues. In agreement with this hypothesis, sign-tracking was nearly doubled when the sole output pathway of the Hb was lesioned. In accordance with the associative opponent model of appetitive-aversive conditioning, in which appetitive and aversive motivational systems are in linked opposition (Solomon and Corbit, 1974), the removal of a tonic Hb-driven inhibitory bias on DA neurons would be expected to diminish constraints on the appetitive salience attributed to reward predicting cues. The role of the Hb in governing the attribution of incentive salience is further supported by the results obtained using a partial-reinforcement schedule that required scaling between two CS’s rather than an all or nothing categorization. In the sham group, substituting the CS- with a partially reinforced cue (CS^33^) initially resulted in approach to both the CS+ and CS^33^. However, a clear bias in the sign-tracking toward the CS+ was observed across sessions. Removing Hb output had an unexpected effect on task performance. Lesioned animals initially exhibited a strong preference for the CS+ over the CS^33^, a response pattern very similar to sham rats exposed to the full contrast conditioning schedule. Interestingly, as the conditioning sessions continued, conditioned approach to the CS^33^ increased as approaches to the CS+ declined, eventually merging to levels that were not significantly different. Goal-tracking was not altered by the lesion. Thus, removing Hb output eliminated the incentive salience scaling that occurs during conditioning yet left the predictive value of the cues intact. Unpredictable reward delivery requires greater discrimination of incentive value than a full contrast condition and can actually increase sign-tracking to the less-predictable cue (Anselme et al., 2013). On the other hand, it is well established that in order to achieve discriminated approach in an autoshaping paradigm, a cue must be uniquely predictive of the reward (Egger and Miller, 1962, 1963). By “ungoverning” incentive salience attributed to the CS^33^, lesioned rats would respond as though both cues were equivalent in their incentive value and would not develop conditioned approach to either stimulus, much as we observed in the final sessions of the partial contrast conditioning schedule. In both the full and the partial contrast conditions, the consequences of the lesion grew more prominent as a function of training; this pattern suggests selective involvement in the process that attributes incentive salience.

A unique opportunity to parse the differences between goal tracking and sign-tracking was provided by studies in which the Hb was stimulated during discrete epochs of the autoshaping procedure (reward delivery, US vs. cue presentation, CS+). Had Hb activation during reward delivery diminished the hedonic impact of the reward or mimicked the neural signal that encodes ‘a less than expected reward’ (i.e., negative temporal difference error) a role for the Hb in strict reward prediction learning would have been implied. However, this was not the case as goal-tracking was not altered by stimulation. Rather, *fr* stimulation during presentation of the CS+ diminished sign-tracking as evidenced by a significant reduction in conditioned approach to the CS+. Previous studies have shown that Hb stimulation suppresses operant responding and induced place avoidance (Friedman et al., 2011; Lammel et al., 2012; Stamatakis and Stuber, 2012), changes consistent with an aversive effect. However, if *fr* stimulation were acting merely as an aversive stimulus, stimulation during presentation of the US would have led to a decrease in goal tracking.

Previous studies by Day et al. (2007) and Flagel et al. (2011b) provide a neurochemical framework to contextualize the present findings. These investigators have shown that during the initial autoshape training period, DA release in the NAc is phasically increased during all three components of a full contrast autoshape conditioning schedule (i.e., reward delivery, CS+ and CS- presentation). As training progresses however, DA release is limited to animals in which CS+ presentation gains incentive motivational properties. It is the phasic increase in NAc DA release during CS+ presentation that is thought to be principally responsible for the attribution of incentive salience to the cue. Further support for this is notion is the demonstration of a correlation between the development of a phasic DA response to a cocaine cue and the probability of approaching that cue (Aragona et al., 2009) and that pharmacological blockade of the DA receptor in the core of the NAc decreases the performance of an already learned sign-tracking conditioned response but not goal-tracking (Saunders and Robinson, 2012). It is conceivable that by stimulating Hb efferents during CS+ presentation, we suppressed VTA DA cell firing and by extension, DA release in the NAc (Lecourtier et al., 2008), thereby decreasing the attribution of incentive salience to the CS+. Athough Hb stimulation during CS+ presentation was more effective in disrupting the attribution of incentive salience, smaller changes were also observed in response to stimuli applied during reward delivery. These changes could reflect an attenuation in the increase in DA cell firing needed to stamp-in reward-relevant cues (Wise, 2004) or a decrease in DA-mediated “reboosting” (Berridge, 2007). Overall, the current studies provide evidence to support a role for the Hb and its downstream targets in encoding incentive salience. The relevant circuit likely involves connections with the RMTg, a collection of GABAergic neurons that provide an important source of feedforward inhibitory input to midbrain DA neurons (Jhou et al., 2009).

The attribution of incentive salience is critical for motivation and the pursuit of rewards. Incentive salience attribution endows a reward predicting stimulus with the capacity to initiate goal-directed behavior and, under some conditions, support behavior leading to their presentation (Rescorla and Solomon, 1967; Cools et al., 2007, 2009). Taken together, the results of the present studies support the contention that the Hb is involved in regulating or scaling the relative degree of incentive salience assigned to a given cue. Accordingly, pathological changes in the activity of Hb neurons could result in avolition and depression-like symptoms or the aberrant pursuit of debilitating goals.

## Conflict of Interest Statement

The authors declare that the research was conducted in the absence of any commercial or financial relationships that could be construed as a potential conflict of interest.

## References

[B1] Anselme P. Robinson M. J. Berridge K. C. (2013). Reward uncertainty enhances incentive salience attribution as sign-tracking. *Behav. Brain Res.* 238 53–61 10.1016/j.bbr.2012.10.006 23078951 PMC4066390

[B2] Aragona B. J. Day J. J. Roitman M. F. Cleaveland N. A. Wightman R. M. Carelli R. M. (2009). Regional specificity in the real-time development of phasic dopamine transmission patterns during acquisition of a cue-cocaine association in rats. *Eur. J. Neurosci.* 30 1889–1899 10.1111/j.1460-9568.2009.07027.x 19912327 PMC2945681

[B3] Berridge K. C. (2007). The debate over dopamine’s role in reward: the case for incentive salience. *Psychopharmacology* 191 391–431 10.1007/s00213-006-0578-x 17072591

[B4] Bromberg-Martin E. S. Matsumoto M. Hikosaka O. (2010). Distinct tonic and phasic anticipatory activity in lateral habenula and dopamine neurons. *Neuron* 67 144–155 10.1016/j.neuron.2010.06.016 20624598 PMC2905384

[B5] Brown P. L. Jenkins H. M. (1968). Auto-shaping of the pigeon’s key-peck. *J. Exp. Anal. Behav.* 11 1–8 10.1901/jeab.1968.11-1 5636851 PMC1338436

[B6] Brown P. L. Shepard P. D. (2013). Lesions of the fasciculus retroflexus alter footshock-induced cFos expression in the mesopontine rostromedial tegmental area of rats. *PLoS ONE* 8:e60678. 10.1371/journal.pone.0060678 PMC3625179 23593280

[B7] Cohen A. S. Najolia G. M. Kim Y. Dinzeo T. J. (2012). On the boundaries of blunt affect/alogia across severe mental illness: implications for Research Domain Criteria. *Schizophr. Res.* 140 41–45 10.1016/j.schres.2012.07.001 22831770

[B8] Cools R. Frank M. J. Gibbs S. E. Miyakawa A. Jagust W D’Esposito M. (2009). Striatal dopamine predicts outcome-specific reversal learning and its sensitivity to dopaminergic drug administration. *J. Neurosci.* 29 1538–1543 10.1523/JNEUROSCI.4467-08.2009 19193900 PMC2940719

[B9] Cools R. Lewis S. J. Clark L. Barker R. A. Robbins T. W. (2007). L-DOPA disrupts activity in the nucleus accumbens during reversal learning in Parkinson’s disease. *Neuropsychopharmacology* 32 180–189 10.1038/sj.npp.1301153 16841074

[B10] Danna C. L. Elmer G. I. (2010). Disruption of conditioned reward association by typical and atypical antipsychotics. *Pharmacol.**Biochem. Behav.* 96 40–47 10.1016/j.pbb.2010.04.004 20416333 PMC3752986

[B11] Day J. J. Roitman M. F. Wightman R. M. Carelli R. M. (2007). Associative learning mediates dynamic shifts in dopamine signaling in the nucleus accumbens. *Nat. Neurosci.* 10 1020–1028 10.1038/nn1923 17603481

[B12] Egger M. D. Miller N. E. (1962). Secondary reinforcement in rats as a function of information value and reliability of the stimulus. *J. Exp. Psychol.* 64 97–104 10.1037/h0040364 13889429

[B13] Egger M. D. Miller N. E. (1963). When is reward reinforcing? An experimental study of the information hypothesis. *J. Comp. Physiol. Psychol.* 56 132–137 10.1037/h0040744

[B14] Flagel S. B. Cameron C. M. Pickup K. N. Watson S. J. Akil H. Robinson T. E. (2011a). A food predictive cue must be attributed with incentive salience for it to induce c-fos mRNA expression in cortico-striatal-thalamic brain regions. *Neuroscience* 196 80–96 10.1016/j.neuroscience.2011.09.004. Epub 2011 Sep10 21945724 PMC3206316

[B15] Flagel S. B. Clark J. J. Robinson T. E. Mayo L. Czuj A. Willuhn I. (2011b). A selective role for dopamine in stimulus-reward learning. *Nature* 469 53–57 10.1038/nature09588 21150898 PMC3058375

[B16] Friedman A. Lax E. Dikshtein Y. Abraham L. Flaumenhaft Y. Sudai E. (2011). Electrical stimulation of the lateral habenula produces an inhibitory effect on sucrose self-administration. *Neuropharmacology* 60 381–387 10.1016/j.neuropharm.2010.10.006 20955718 PMC3056985

[B17] Gamzu E. R. Williams D. R. (1973). Associative factors underlying the pigeon’s key pecking in auto-shaping procedures. *J. Exp. Anal. Behav.* 19 225–232 10.1901/jeab.1973.19-225 16811661 PMC1334074

[B18] Herkenham M Nauta W. J. H. (1979). Efferent connections of the habenular nuclei in the rat. *J. Comp. Neurol.* 187 19–48 10.1002/cne.901870103 226566

[B19] Hikosaka O. (2010). The habenula: from stress evasion to value-based decision-making. *Nat. Rev.* 11 503–513 10.1038/nrn2866 PMC3447364 20559337

[B20] Hong S. Jhou T. C. Smith M. Saleem K. S. Hikosaka O. (2011). Negative reward signals from the lateral habenula to dopamine neurons are mediated by rostromedial tegmental nucleus in primates. *J. Neurosci.* 31 11457–11471 10.1523/JNEUROSCI.1384-11.2011 21832176 PMC3315151

[B21] Insel T. Cuthbert B. Garvey M. Heinssen R. Pine D. S. Quinn K. (2010). Research domain criteria (RDoC): toward a new classification framework for research on mental disorders. *Am. J. Psychiatry* 167 748–751 10.1176/appi.ajp.2010.09091379 20595427

[B22] Jesuthasan S. (2012). Fear, anxiety, and control in the zebrafish. *Dev. Neurobiol.* 72 395–403 10.1002/dneu.20873 22328274

[B23] Jhou T. C. Geisler S. Marinelli M. Degarmo B. A. Zahm D. S. (2009). The mesopontine rostromedial tegmental nucleus: a structure targeted by the lateral habenula that projects to the ventral tegmental area of Tsai and substantia nigra compacta. *J. Comp. Neurol.* 513 566–596 10.1002/cne.21891 19235216 PMC3116663

[B24] Jhou T. C. Good C. H. Rowley C. S. Xu S. P. Wang H. Burnham N. W. (2013). Cocaine drives aversive conditioning via delayed activation of dopamine-responsive habenular and midbrain pathways. *J. Neurosci.* 33 7501–7512 10.1523/JNEUROSCI.3634-12.2013 23616555 PMC3865501

[B25] Ji H. Shepard P. D. (2007). Lateral habenula stimulation inhibits rat midbrain dopamine neurons through a GABA_A_ receptor-mediated mechanism. *J. Neurosci.* 27 6923–6930 10.1523/JNEUROSCI.0958-07.2007 17596440 PMC6672239

[B26] Lammel S. Lim B. K. Ran C. Huang K. W. Betley M. J. Tye K. M. (2012). Input-specific control of reward and aversion in the ventral tegmental area. *Nature* 491 212–217 10.1038/nature11527 23064228 PMC3493743

[B27] Lecourtier L. Defrancesco A. Moghaddam B. (2008). Differential tonic influence of lateral habenula on prefrontal cortex and nucleus accumbens dopamine release. *Eur. J. Neurosci.* 27 1755–1762 10.1111/j.1460-9568.2008.06130.x 18380670 PMC2881677

[B28] Matsumoto M. Hikosaka O. (2007). Lateral habenula as a source of negative reward signals in dopamine neurons. *Nature* 447 1111–1115 10.1038/nature05860 17522629

[B29] Matsumoto M. Hikosaka O. (2009). Representation of negative motivational value in the primate lateral habenula. *Nat. Neurosci.* 12 77–84 10.1038/nn.2233 19043410 PMC2737828

[B30] Morris S. E. Cuthbert B. N. (2012). Research domain criteria: cognitive systems, neural circuits, and dimensions of behavior. *Dialogues Clin. Neurosci*. 14 29–37 22577302 10.31887/DCNS.2012.14.1/smorris PMC3341647

[B31] Parkinson J. A. Willoughby P. J. Robbins T. W. Everitt B. J. (2000). Disconnection of the anterior cingulate cortex and nucleus accumbens core impairs Pavlovian approach behavior: further evidence for limbic cortical-ventral striatopallidal systems. *Behav. Neurosci.* 114 42–63 10.1037/0735-7044.114.1.42 10718261

[B32] Paxinos G. Watson C. (2007). *The rat brain in stereotaxic coordinates* Boston: Elsevier 10.1016/0165-0270(80)90021-7 6110810

[B33] Pellegrini S. Lopez Seal M. F. Papini M. R. (2008). Scaling relative incentive value: different adjustments to incentive downshift in pigeons and rats. *Behav. Processes* 79 182–188 10.1016/j.beproc.2008.07.008 18755254

[B34] Rescorla R. A. Solomon R. L. (1967). Two-process learning theory: relationships between Pavlovian conditioning and instrumental learning. *Psychol. Rev.* 74 151–182 10.1037/h0024475 5342881

[B35] Saunders B. T. Robinson T. E. (2012). The role of dopamine in the accumbens core in the expression of Pavlovian-conditioned responses. *Eur. J. Neurosci.* 36 2521–2532 10.1111/j.1460-9568.2012.08217.x 22780554 PMC3424374

[B36] Shelton L. Pendse G. Maleki N. Moulton E. A. Lebel A. Becerra L. (2012). Mapping pain activation and connectivity of the human habenula. *J. Neurophysiol.* 107 2633–2648 10.1152/jn.00012.2012 22323632 PMC3362277

[B37] Solomon R. L. Corbit J. D. (1974). An opponent-process theory of motivation. I. Temporal dynamics of affect. *Psychol. Rev.* 81 119–145 10.1037/h0036128 4817611

[B38] Stamatakis A. M. Stuber G. D. (2012). Activation of lateral habenula inputs to the ventral midbrain promotes behavioral avoidance. *Nat. Neurosci.* 15 1105–1107 10.1038/nn.3145 22729176 PMC3411914

[B39] Weiss T. Veh R. (2011). Morphological and electrophysiological characteristics of neurons within identified subnuclei of the lateral habenula in rat brain slices. *Neuroscience* 172 74–93 10.1016/j.neuroscience.2010.10.047 20974229

[B40] Wise R. A. (2004). Dopamine, learning and motivation. *Nat. Rev.* 5 483–494 10.1038/nrn1406 15152198

